# Bottom-up reconstitution of model membrane systems: a mini-review

**DOI:** 10.1042/BST20250291

**Published:** 2026-07-16

**Authors:** Hemraj Meena, Katon Wibowo, Nicola De Franceschi

**Affiliations:** IMol Polish Academy of Sciences, 02-247 Warsaw, Poland

**Keywords:** giant liposome, membrane, reconstitituion, supported lipid bilayer, transmembrane proteins

## Abstract

Bottom-up membrane reconstitution has become a powerful framework to investigate the physical principles governing membrane organization and function in highly controlled environments. Among the broad range of available model systems, supported lipid bilayers (SLBs) and giant unilamellar vesicles (GUVs) have emerged as particularly versatile platforms compatible with live optical imaging. In the present mini-review, we summarize recent advances in the preparation and application of these micron-scale membrane systems. We discuss how SLBs and GUVs have enabled major insights into membrane-associated protein assembly, membrane curvature sensing and remodeling, permeability, and cytoskeletal organization. We further highlight emerging developments, including suspended membranes, membrane–coacervate interactions, and the use of GUVs as chassis for bottom-up synthetic biology. Finally, we discuss future perspectives for the field, particularly the growing effort in interfacing synthetic membrane systems and living cells, with potential applications in biomedicine.

## Introduction

Membranes are indispensable to life, acting as dynamic barriers that separate cells from their environment while enabling selective transport, signal transduction, and intercellular communication. Beyond compartmentalization, membranes participate in essential processes including energy conversion, protein synthesis, and intracellular trafficking. Their remarkable versatility arises from a combination of chemical and physical properties that distinguish them from other biological assemblies.

Although membrane thickness remains relatively constant at ≈5 nm, membranes can span lateral dimensions ranging from tens of nanometers to centimeters [1]. Unlike covalent polymers, membranes are supramolecular structures stabilized by non-covalent interactions. Lipid bilayers self-assemble through the hydrophobic effect, driven by the energetic penalty associated with exposing hydrophobic lipid tails to water [2]. Consequently, membranes spontaneously form closed structures that minimize energetically unfavorable edges.

This non-covalent organization endows membranes with unique properties. They can grow through lipid insertion, deform in response to internal or external forces, and undergo fusion and fission. These behaviors underpin key cellular processes, including cell division, vesicular transport, and endocytosis. While lipid composition modulates certain properties such as bending rigidity, degree of deformability and specific affinity to proteins, other features, such as responsiveness to osmotic gradients, largely transcend the chemical composition of the membrane and emerge as general physical characteristics.

The intrinsic ability to self-assemble and the stability of lipid bilayers make membranes particularly amenable to bottom-up reconstitution. Such approaches enable the reconstruction of simplified systems *in vitro*, allowing precise control over composition and environmental conditions. The choice of reconstitution strategy is therefore guided primarily by the specific biophysical or biological question under investigation. A broad range of *in vitro* membrane systems has been developed to investigate membrane organization and function. In the present mini-review, we focus on micron-scale models compatible with live imaging by conventional light microscopy, namely supported lipid bilayers (SLBs) and giant unilamellar vesicles (GUVs). We outline the main methodologies for their preparation, highlight recent technical advances, and discuss their applications, with an emphasis on emerging developments in the field.

## Supported lipid bilayers

SLBs are model membranes with planar configuration generated on top of solid substrates. Developed several decades ago, SLBs have become indispensable tools in membrane biophysics. The most widely used approach for SLB formation is vesicle fusion: small unilamellar vesicles (SUVs), typically 25–100 nm in diameter, adsorb onto hydrophilic substrates such as SiO_2_ or mica, followed by vesicle deformation, rupture, and bilayer spreading [3]. Successful bilayer formation depends on vesicle composition, surface chemistry, ionic strength (particularly Ca^2+^ concentration), and temperature relative to the lipid phase transition temperature (T_m_). An alternative approach, particularly suitable for charged lipids and complex lipid mixtures, is solvent-assisted lipid bilayer formation, which enables SLB assembly during continuous solvent exchange [4].

**Figure 1:**
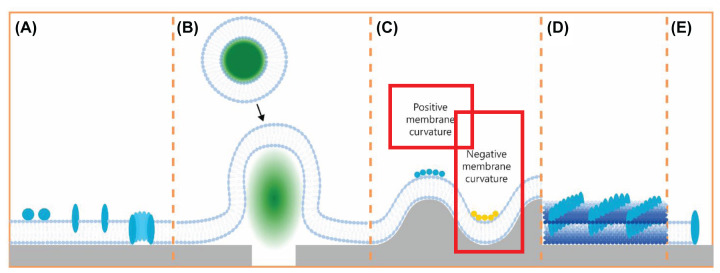
Applications of SLBs to study binding, organization, and membrane remodeling (**A**) Proteins can bind to SLBs with different degrees of penetration, retaining their ability to diffuse on the membrane plane. In contrast, pore-forming proteins such as Gasdermins fully penetrate the bilayer, creating a water channel and becoming immobilized on the SLB support. (**B**) SLBs can be generated on discontinuous supports, obtaining pore-spanning SLBs. These can be used to study vesicle fusion and content release (indicated in green) thanks to the presence of an underlying reservoir. (**C**) SLBs can be generated on non-planar substrates, obtaining membranes with defined curvature. This can be used to characterize binding and assembly of curvature-sensing proteins, which tend to enrich on specific regions of the SLB. In the schematic, blue and yellow proteins preferentially bind to positively and negatively curved membrane, respectively. (**D**) Thanks to their large and uniform surface, SLBs allow visualization and manipulation of nematic order of proteins filaments. (**E**) The SLB edge can be recognized by proteins that either repair or disrupt membranes.

The most common application of SLBs is the study of protein binding and membrane-associated protein complex formation [5,6] (Figure 1A). This approach has proven particularly useful for investigating cytoskeletal proteins such as actin, including how membrane lipid composition organizes cytoskeletal networks and modulates contractile properties at the membrane interface [7]. However, numerous variants of this basic assay have been developed. In one notable example, SLBs were used to investigate the pore-forming mechanism of Gasdermin D: proteins able to fully cross the bilayer were immobilized by the underlying glass support, providing indirect evidence for complete membrane penetration and pore formation [8] (Figure 1A). SLBs are also compatible with super-resolution microscopy, enabling the characterization of nanometer-scale membrane domains [9] and receptor clustering [10]. Moreover, recent technical advances allow the generation of asymmetric SLBs with controlled distributions of specific lipids, particularly phosphoinositides [11], further expanding the versatility of these systems. These examples highlight SLBs as versatile platforms to characterize membranes and membrane-associated proteins with high spatial resolution. Beyond these ‘classical’ binding assays, numerous creative applications of SLBs have emerged.

### Pore-spanning membranes

A direct consequence of conventional SLB formation is that the bilayer remains in close contact with the solid support, which reduces lipid mobility and can interfere with membrane remodeling processes such as reshaping, pore formation, and vesicle fusion. One solution to this problem is generating partially free-standing SLBs by rupturing GUVs on substrates containing micrometer-size pores. This results in suspended membrane regions with a more physiological mechanical state, enabling improved investigation of protein and lipid organization [12] as well as direct observation of dynamic membrane remodeling events beyond simple protein binding. For example, this approach enabled direct visualization of SNARE-mediated vesicle fusion and content release [13] (Figure 1B). Pore-spanning membranes can also be generated using native membranes, further broadening the scope of this technique [14].

### SLBs built on non-planar and soft surface

Building lipid bilayers on solid supports allows membrane geometry to be modulated through the use of custom-designed substrates. This is particularly useful for investigating membrane geometries that are transient or difficult to reproduce *in vitro*, such as regions of negative curvature (Figure 1C). SLBs assembled on non-planar surfaces also offer the advantage of using repetitive topographical features, thereby reproducing defined membrane geometries over large areas and making the approach relatively high-throughput. This technique has mainly been used to generate undulated or conical membrane features with characteristic sizes ranging from a few micrometers down to ≈100 nm [15,16].

Using undulated SLBs, septin filaments were shown to sense and therefore enrich on negatively curved membrane regions while orienting perpendicularly to the membrane undulations, thereby maximizing curvature sensing [17]. These findings confirm earlier observations on septin curvature sensitivity [15]. Interestingly, increasing protein concentration led to the formation of two perpendicular septin layers [17]. This arrangement resembles the organization previously observed for ESCRT-III proteins [18], suggesting that such higher-order architectures may represent a more general feature of cytoskeletal filament systems rather than a property specific to septins.

On SLBs deposited onto substrates containing periodic conical protrusions, the ESCRT-III protein Snf7 assembled into spirals displaying emergent curvature sensitivity, adapting their organization to local membrane geometry while minimizing internal filament stress. It was further demonstrated that mechanical energy stored within the filament can drive spiral buckling and membrane remodeling [16]. Importantly, these observations relied on the ability to generate SLBs on deformable polydimethylsiloxane (PDMS) substrates, which allowed membrane remodeling along the *z*-axis. Beyond illustrating the versatility of SLBs for probing membrane curvature, these studies also demonstrate the compatibility of SLBs with multiple imaging modalities, including atomic force microscopy, transmission electron microscopy, and conventional light microscopy.

### SLB to visualize nematic order of filaments

Because of their large continuous surface, SLBs are particularly well suited to investigate the mesoscale organization of cytoskeletal filaments, such as their nematic order (Figure 1D). Septin filaments were shown to display both lateral and longitudinal alignment, preferentially on liquid-disordered membrane phases [19]. Interestingly, disruption experiments using controlled AFM tip forces demonstrated that these ordered septin assemblies could recover through self-templating mechanisms, suggesting that the pre-existing filament network encodes structural information that stabilizes higher-order organization. An interesting extension of this approach involved investigating the nematic organization of vimentin intermediate filaments upon stretching of an SLB generated on an elastic PDMS substrate, where excess membrane area was supplied by membrane reservoirs. Individual vimentin filaments reorganized and elongated along the direction of stretching, whereas dense filament networks primarily underwent collective reorientation rather than significant elongation. These observations reveal that membrane-associated intermediate filaments are mechanically responsive and dynamically adapt their architecture in response to external forces, suggesting a possible role in cortical mechanotransduction, where cytoskeletal elements near the plasma membrane contribute to sensing and responding to mechanical stress. This work highlights the interplay between membrane mechanics and filament dynamics, and provides a model for understanding how intermediate filaments contribute to cellular structural integrity under deformation [20].

### SLB edge as a tool to study protein binding

Whereas free-standing membranes spontaneously close to avoid exposed edges, the strong interaction between SLBs and their supporting substrate permits open membrane edges to persist for a prolonged time. These edges recapitulate structures transiently generated during membrane rupture or damage. Such traumatic events can have severe cellular consequences, ranging from DNA leakage following nuclear envelope rupture [21] to cell death induced by plasma membrane disruption [22]. Cells have therefore evolved dedicated protein machineries, notably the ESCRT-III complex, to resolve these potentially lethal defects. Due to their highly dynamic and catastrophic nature, membrane rupture events are difficult to study *in vitro*. SLBs provide a powerful platform to address this challenge because membrane edges can remain stable for extended periods (Figure 1E). This concept has been exploited to characterize the membrane-disrupting activity of microbial peptides [23,24] and to investigate how ESCRT proteins sense highly curved membrane edges and promote membrane sealing [25,26].

## Giant unilamellar vesicles

GUVs are closed membrane structures composed of a single lipid bilayer enclosing an aqueous lumen and are formally defined by diameters larger than 1 μm. Three main methodologies are commonly used for GUV production, each relying on distinct biophysical mechanisms. The oldest approach is based on spontaneous swelling of a dry lipid film deposited on a surface. GUV yield and degree of unilamellarity strongly depend on both the chemical and physical properties of the substrate and the lipid composition. Over the years, two major improvements to this technique have emerged. The gel-assisted method employs a polymer layer, such as polyvinyl alcohol, to enhance vesicle swelling [27]. Electroformation, in contrast, uses alternating electric fields to promote vesicle swelling from conductive glass slides or platinum wires coated with lipids [28]. Both gel-assisted swelling and electroformation are relatively straightforward to implement and substantially reduce the formation of multilamellar vesicles while increasing overall GUV yield. Moreover, both methods have been used for the functional reconstitution of transmembrane proteins [29–31], although this generally requires extensive case-by-case optimization. However, all swelling-based approaches suffer from poor encapsulation efficiency of macromolecules within the GUV lumen, as well as reduced yields in high-salt buffers or in the presence of large fractions of charged lipids.

GUVs can also be generated using the inverted emulsion method, also known as droplet transfer. In this approach, a small volume of aqueous solution is emulsified within an organic phase containing lipids dissolved in oil. The resulting micrometer-sized droplets become stabilized by the spontaneous assembly of a lipid monolayer at the oil–water interface. Subsequently, the droplets cross a second oil/outer-buffer interface, where they acquire a second lipid monolayer to form complete bilayers [32]. A major advantage of this method is its high encapsulation efficiency for macromolecules within the GUV lumen, along with its compatibility with methodologies that allows continuous production, such as microfluidics [33] or cDICE [34]. Limitations include inefficient incorporation of lipids with high chain-melting temperatures and cholesterol, as well as the formation of lipid aggregates on the GUV membrane. This latter issue, however, has recently been solved by optimizing the composition of the organic phase [35].

A third approach for GUV generation is based on fusion of pre-existing membranes or membrane fragments to a micrometer-sized template. Both SUVs and nanodiscs or nanoparticles have been explored as membrane source. This approach is particularly promising because it combines efficient encapsulation with the possibility of functionally reconstituting transmembrane proteins [36–38], which remains technically challenging with other GUV preparation methods. One limitation of using nanodiscs or nanoparticles as the sole membrane source is increased membrane leakiness [38]. An interesting recent innovation is the combination of inverted emulsion and nanoparticles to simultaneously achieve both proteoGUVs generation and controlled membrane deformation in a single step [39].

**Figure 2:**
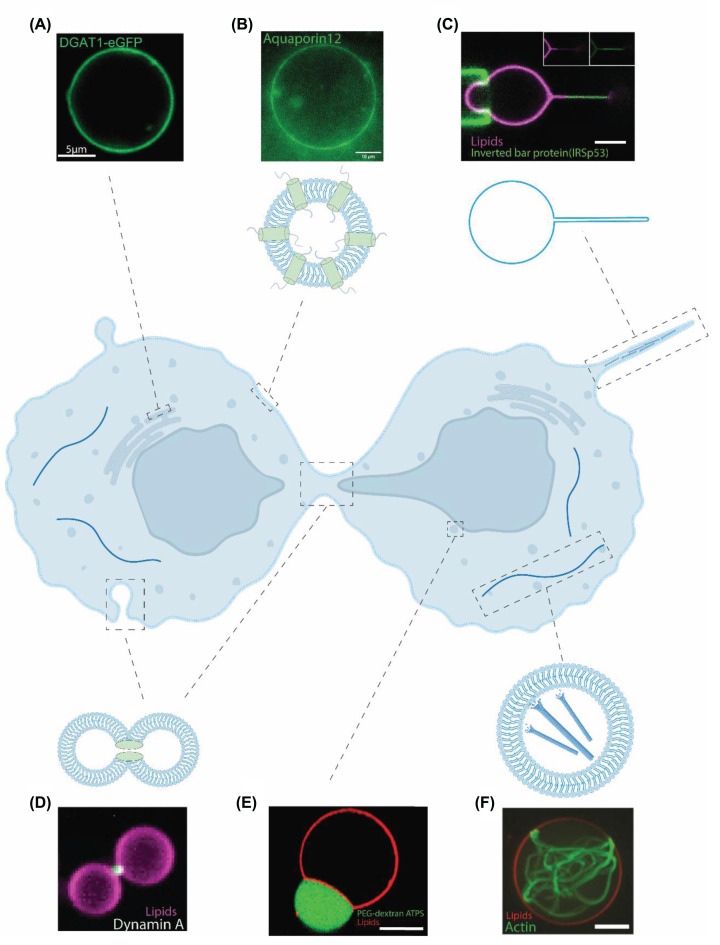
Applications of GUVs to study protein function and membrane remodeling (**A**) Generation of GOVs from organelles allows to retain the original lipid and protein composition of the organelle in order to characterize their biophysical properties. (**B**) Transmembrane proteins and pores, such as Aquaporin, can be reconstituted in GUVs to characterize membrane transport dynamically using optical microscopy. (**C**) Nanotube pulling assay allows to study how proteins sense and generate membrane curvature. (**D**) Using the synthetic membrane shaper (SMS) approach, reconstitution of curvature-sensing proteins at the neck of dumbbell-shaped GUVs allowed to reconstitute cell division *in vitro*. Similar membrane geometries are present throughout the cell, for instance during the process of endocytosis. (**E**) Interaction between coacervates and membranes can result in mutual remodeling, with physiological and pathological implications that are just beginning to be unveiled. (**F**) Reconstitution of cytoskeletal networks inside GUVs are useful to characterize how cytoskeleton impacts the shape and mechanical properties of the membrane. Images in panels (A),(C–F) were adapted from [40,41,42–44].

Cellular plasma membranes can also serve as membrane sources. Induction of cell blebbing generates giant plasma membrane vesicles, which preserve several physiological membrane properties that are difficult to reproduce using bottom-up reconstitution approaches, including native compositional complexity, lipid asymmetry, and endogenous protein content. Until recently, such cell-derived giant vesicles were largely restricted to plasma membranes. However, osmotic pressure-driven vesiculation has recently enabled the production of giant vesicles derived from intracellular organelles, termed giant organelle vesicles (GOVs). GOVs retain lipid and protein composition of their source organelles, resulting in distinct elasto-mechanical properties depending on the organelle of origin [40] (Figure 2A).

Taking advantage of their size, GUVs have been extensively used to visualize protein binding, probe membrane permeability, investigate the effects of membrane curvature, as well as chassis for bottom-up synthetic biology. Here, we review the most recent advancements in these applications.

### Membrane permeability

Investigating membrane permeability remains an active field of research, with pore-forming activity of proteins and peptides continuously being characterized [45,46] (Figure 2B). In parallel, a broad range of synthetic pores has been developed, primarily using nucleic acid nanotechnology and subsequently tested in GUVs. These synthetic pores can match, and in some cases exceed, the dimensions of naturally occurring protein pores [47,48]. A key advantage of nucleic acid nanostructures lies in the possibility of engineering and precisely controlling their activity, including the ability to trigger pore formation in response to external stimuli [49] and to finely tune pore size [50].

### Membrane curvature

GUVs are powerful tools to investigate how membrane curvature influences protein binding, sorting, and function, including membrane remodeling and scission. Traditionally, these questions have been addressed using tube-pulling techniques, in which membrane nanotubes are mechanically extracted from GUVs, generating highly curved membrane necks. Tube pulling enables simultaneous quantification of curvature-dependent protein sorting and membrane forces generated by proteins themselves, but remains technically very demanding [51,52] (Figure 2C). Recently, an alternative approach enabling higher throughput and easier generation of curved membrane geometries has been introduced. The SMS technique allows the production of large numbers of dumbbell- or stomatocyte-shaped GUVs featuring catenoid necks. Proteins can be reconstituted on either side of the catenoid neck, thereby having access to a range of different curvatures [41]. This system has been used to study recruitment of archaeal Cdv proteins [53] and to directly visualize membrane scission mediated by bacterial dynamin A [54] (Figure 2D). The SMS is also compatible with transmembrane proteins reconstitution [39]. However, unlike the tube-pulling approach, SMS does not provide direct measurements of membrane tension.

### Membrane–coacervates interaction

Recently, a new class of membrane interactions involving biomolecular condensates has emerged (Figure 2E). Liquid–liquid phase separation is now recognized as a major organizing principle in cell biology [55,56]. Interactions between membranes and phase-separated droplets, also referred to as coacervates, are increasingly being recognized as important regulatory mechanisms [57] involved in both physiology and pathology [58,59]. These interactions can drive reciprocal remodeling between membranes and condensates, yet remain difficult to study *in vivo* because of the small size and highly dynamic nature of coacervates, together with the crowded intracellular environment. Bottom-up reconstitution is therefore particularly well suited to investigate these processes. Depending on their affinity for membranes, coacervates can alter lipid packing and induce membrane curvature, leading to the formation of membrane nanotubes and sheets [60]. A striking demonstration of the biological relevance of these interactions was the observation that coacervates can plug micrometer-sized holes in GUVs, mimicking the stabilizing role of stress granules during endolysosomal membrane damage. Coacervate presence facilitated membrane repair, potentially through spontaneous engulfment driven by membrane wetting of the condensate surface [61].

### Chassis for synthetic biology

Over the past few years GUVs have emerged as the preferred chassis for what is commonly referred to, somewhat generously, as synthetic cells. These are excellent models to reconstitute cellular components such as cytoskeletal networks, as well as processes including transport and division (Figure 2F). The cytoskeleton plays a central role in cellular organization by providing mechanical stability while enabling vesicular trafficking, force generation, and signal transduction [62]. Reconstitution of actin filaments inside GUVs, along with the presence of cross-linkers and engineered membrane attachment resulted in the formation of of membrane-associated actin rings. Furthermore, addition of myosin II resulted in ring contraction [63]. Subsequent work expanded this framework by reconstituting the bacterial MinD/MinE system together with actomyosin networks inside GUVs. Through diffusio-phoretic coupling between the Min protein gradients and actomyosin, contractile rings were stabilized at the midplane of the vesicles, leading to furrow-like membrane deformations reminiscent of early cell division events. These fascinating studies represent key milestones in bottom-up synthetic biology, illustrating how spatial organization and mechanical function can emerge from minimal sets of components [64].

Beyond cytoskeletal systems, reconstitution of signal transduction modules within GUVs has gained increasing attention. Recent reviews highlight progress in constructing mechanosensitive and biochemical signaling pathways *de novo*, further emphasizing the versatility of GUVs based system for dissecting complex cellular behaviors in a controlled environment [65].

One of the current frontiers in synthetic biology is the interfacing of biological and synthetic membrane-based systems with the aim of restoring lost cellular functions or introducing new functionalities. Examples include the creation of hybrid tissues such as lymphatic organs that support *ex vivo* expansion of immune cells [66] and the development of artificial tumor immune microenvironments [67].

## Perspectives

- **The importance of the field:** Bottom-up membrane reconstitution has now matured into a well-established field. While new variations of the methodologies described here continue to emerge, a broad range of robust approaches is already available to characterize membranes and their interactions with macromolecules. Over the past decades, fundamental insights have been gained into how membrane properties such as fluidity, curvature, and tension, together with environmental parameters including osmotic pressure influence membrane shape, protein assembly, and membrane remodeling. These studies now provide a general framework for understanding many cellular processes that rely on membrane dynamics.
- **Summary of the current thinking:** Membrane-associated protein assembly and processes such as membrane remodeling and permeabilization remain highly active areas of investigation. At the same time, interactions between membranes and biomolecular condensates have recently emerged as a particularly exciting and rapidly developing topic. Given the growing recognition of membrane–coacervate interactions in both physiology and pathology, this area is likely to remain at the forefront of bottom-up membrane reconstitution studies in the coming years.
- **Future directions:** The knowledge gained from bottom-up membrane reconstitution is also highly relevant for applications in drug delivery and biomedicine more broadly. In this context, interfacing synthetic membrane-based systems with living systems is likely to become an important direction for future research. In contrast to the ambitious goal of constructing fully autonomous synthetic cells, which remains technically extremely challenging, the development of low-complexity synthetic systems designed for specific tasks appears more feasible in the near term. However, successful and reproducible integration of even limited numbers of biological components into such systems—and, importantly, the ability to produce them at scale—will require the development of robust and scalable methodologies, or substantial improvements to existing ones. While *ex vivo* coexistence between synthetic and natural cells has already been demonstrated, the integration of such complex synthetic systems *in vivo* remains challenging, notably because of host immune responses. Overcoming these limitations will be essential for these technologies to fully realize their biomedical potential.

## Open Access

This article has been published open access under our Subscribe to Open programme, made possible through the support of our subscribing institutions, learn more here: https://portlandpress.com/pages/open_access_options_and_prices#conditional.

## Abbreviations

GOV: giant organelle vesicles
GUV: giant unilamellar vesicle
PDMS: polydimethylsiloxane
SLB: supported lipid bilayer
SMS: synthetic membrane shaper
SUV: small unilamellar vesicle
